# Acute Chest Pain Outside Pulmonary Embolism: Diagnosis and Management

**DOI:** 10.5334/jbr-btr.1221

**Published:** 2016-11-19

**Authors:** Rodrigo Salgado

**Affiliations:** 1 Dept. of Radiology, Antwerp University Hospital, Antwerp, Belgium

**Keywords:** dissection, intramural hematoma, penetrating atherosclerotic ulcer, acute chest pain, CT angiography, coronary arteries, aortic trauma

## Introduction

Acute chest pain is a common emergency department presentation. Several causes are known with varying degrees of clinical importance, ranging from hyperventilation to a ruptured thoracic aneurysm. As clinical signs and symptoms are non-specific, imaging is often requested in the diagnostic work-up. The focus in this review will be placed on acute pathology emerging from the thoracic aorta and the coronary arteries, with emphasis on the role of non-invasive computed tomography (CT) and magnetic resonance imaging (MRI) in the triage of these patients.

## Acute Aortic Pathology

Acute aortic events are often summarized under the term ‘acute aortic syndrome’. This syndrome encompasses classic aortic dissection, intramural hematoma and penetrating atherosclerotic ulcer. Its incidence ranges from 3.5 to 6.0 per 100,000 patients per year in the United States, but is higher in older patients, especially after the age of 75 years [1,2]. While the distinction between these three entities continues to be used in the scientific literature, it has come under criticism; according to some authors it mixes distinct clinical entities (like a classic aortic dissection) with disease presentations (like intramural hematoma) which are maybe better conceptualized as an imaging finding rather than an actual disease [3,4]. Also, while an aortic dissection and intramural hematoma are pathologies arising from the aortic media layer, a penetrating atherosclerotic ulcer has a completely different aetiology, but can also lead to an intramural hematoma [5].

### Acute aortic dissections

About 80–90% of all acute aortic syndromes are aortic dissections [6]. Although many causes have been identified as causing an aortic dissection, presence of arterial hypertension is one of the most encountered risk factors, observed in 45% to 100% of patients with an acute aortic dissection, followed by smoking in 20% to 85% [7].

Aortic dissections can be classified using either the DeBakey classification or Stanford classification.

The DeBakey classification distinguishes 3 different types of dissection. Type I involves the ascending aorta, the arch, and a variable length of the thoracic and abdominal aorta. A type II dissection is restricted to the ascending aorta, while a type IIIA dissection is confined to the descending thoracic aorta. Finally, in type IIIB the dissection extends into the abdominal aorta and the iliac vessels.

**Figure 1:**
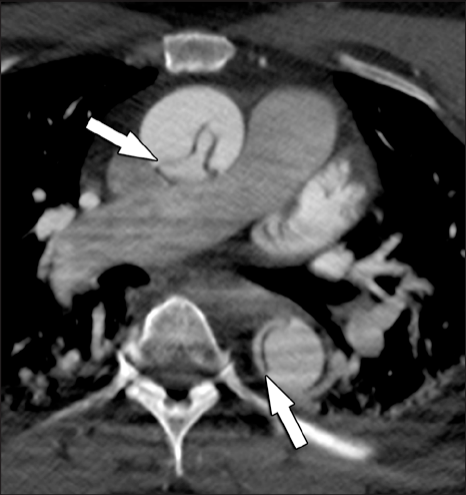
A classic type-A dissection in a 53-year-old man with known arterial hypertension. The dissection flap is clearly seen in the ascending and descending aorta (arrows). The presence of a dissection flap in the ascending aorta classifies this as a Stanford type-A aortic dissection.

**Figure 2:**
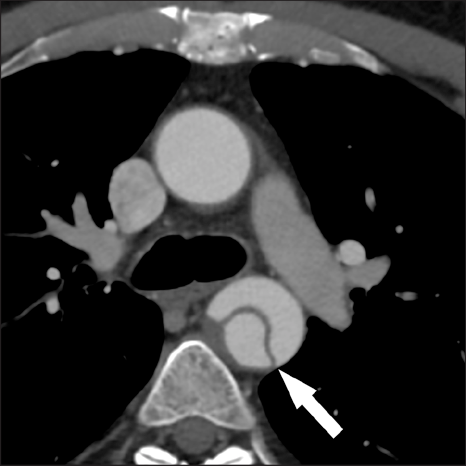
Type-B aortic dissection in a 65-year-old man. Note the dissection flap in the descending aorta (arrow), with no involvement of the ascending aorta.

Conversely, the Stanford classification only distinguishes between two types: type A and B aortic dissection. A type A dissection is defined as involving the ascending aorta, regardless of further involvement of the aortic arch and descending aorta (Figure 1). A type-B dissection includes every dissection in which the ascending aorta is not involved, and is typically used to indicate a dissection starting after the origin of the left subclavian artery (Figure 2).

An acute aortic dissection always starts with an initial rupture of the inner intima wall of the aorta. Through this ‘entry’ point, blood from the lumen can flow in the media layer of the aortic wall, splitting it in a longitudinal fashion and forming a dissection flap. As such, two channels are created: the original ‘true’ lumen of the aorta, and a secondary created ‘false’ lumen within the contour of the aorta. Note that the dissection flap is composed of both intima and to a lesser extend media components, leaving a weakened wall along the false lumen side.

Many centres routinely use the Stanford classification for the distinction between a dissection with its origin proximal or distal from the ostium of the left subclavian artery. This distinction quickly differentiates between different treatment strategies (in general urgent surgery for type A dissection vs. strict hypertension control and follow-up for uncomplicated type B dissections).

### Intramural hematoma

Traditionally, an intramural hematoma has for long been believed to be the consequence of a de novo bleeding in the media layer of the aortic wall, without communication with the true aortic lumen.

**Figure 3:**
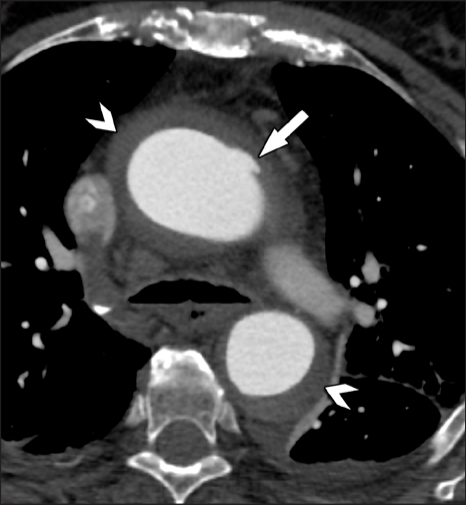
A small defect in the intimal aortic layer (arrow) can be seen, representing an entry point for an intramural hematoma extending into the ascending and descending aorta (arrowheads).

As imaging techniques evolve, this etiology has been questioned, as in some cases small intimomedial tears have been identified (Figure 3) [4,8]. It is now more appropriately conceptualised by some as having the same origins as a classic dissection, and as such represents a different presentation of the same disease arising from a diseased aortic media [5,8]. The fundamental thought is here to conceptualise the true and false lumen in an aortic dissection as two separate channels, which may or may not both contain flowing blood.

In a classic aortic dissection, these two channels both contain flowing blood along the course of the dissection, which typically ends with a re-entry point where the blood in the false lumen re-enters the original vessel lumen. The original true lumen contains fast flowing blood, while the false lumen contains slower flowing blood. This slower blood flow contributes to the forming of thrombus along the border of the false lumen, typically seen in segments with larger diameter.

**Figure 4:**
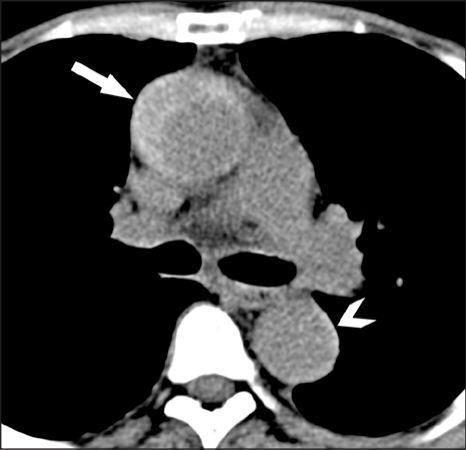
Unenhanced CT of the aorta in a 58-year-old woman with acute chest pain. A spontaneous dense semicircular structure can be seen in the ascending aorta, representing an acute intramural hematoma with wall thrombus (arrows in A-B). A small extension of this intramural hematoma can also be seen in the descending aorta (arrowhead in A).

Following this two-channel model, an intramural hematoma can be best understood as a situation where there is only one channel of flowing blood (the true lumen), and one channel of stationary blood (the thrombosed false lumen). This can occur in cases where no clear exit or re-entry site is present for the false lumen, leading to circulatory stasis and thrombus formation of the false lumen. On unenhanced CT-images this intramural hematoma can be seen in the acute stage as a spontaneous dense semicircular thickening of the aortic wall along a segment of variable length (Figure 4). After intravenous contrast administration, this high-density appearance is less obvious, making it sometimes more difficult to appreciate.

**Figure 5:**
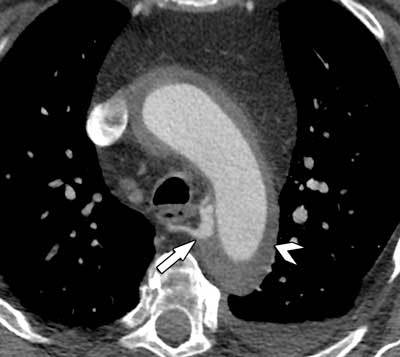
An intramural blood pool (arrow) can be seen, feeding from an intercostal artery. This small blood collection appears in an otherwise thrombosed intramural hematoma (arrowhead).

Within the thrombosed false lumen, small intramural blood pools can occasionally be seen (Figure 5). These small enhancing areas within the intramural hematoma are the result of retrograde filling from intercostal arteries, and do not represent small communications with the enhancing lumen. Over time, these intramural blood pools can remain stable, regress or progress in size.

**Figure 6:**
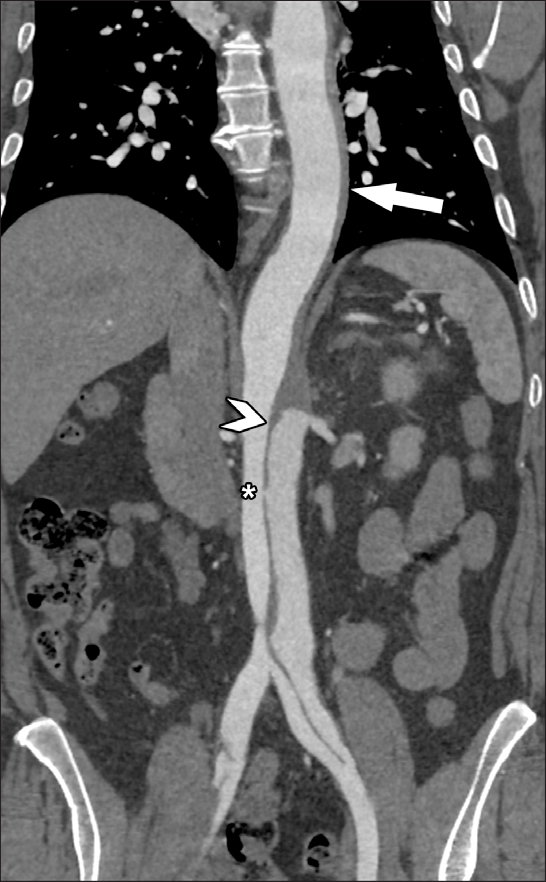
Simultaneous presence of an intramural hematoma in the descending thoracic aorta (arrow), changing into a classic aortic dissection in the abdominal aorta (arrowhead) in a 61-year-old woman. An intramural hematoma can always evolve into a classic aortic dissection, as small ruptures and fenestrations (asterisk) within the aortic wall change the hemodynamics in the true and false lumina.

It is important to realise that an intramural hematoma is clinically undistinguishable from a classic dissection, can affect both the ascending and descending aorta as a type-A dissection or starting after the left subclavian artery origin like a classic type-B dissection. Therefore, an intramural hematoma is handled identically by many centres and treated as a classic dissection, and it can often be found together with a classic aortic dissection within the same patient (Figure 6).

However, small intramural hematomas have been known to resorb over time without surgical intervention.

### Penetrating atherosclerotic ulcer

A penetrating atherosclerotic ulcer is defined as an atherosclerotic ulceration, penetrating from a diseased thickened intima layer through the internal elastic lamina into the aortic media.

**Figure 7:**
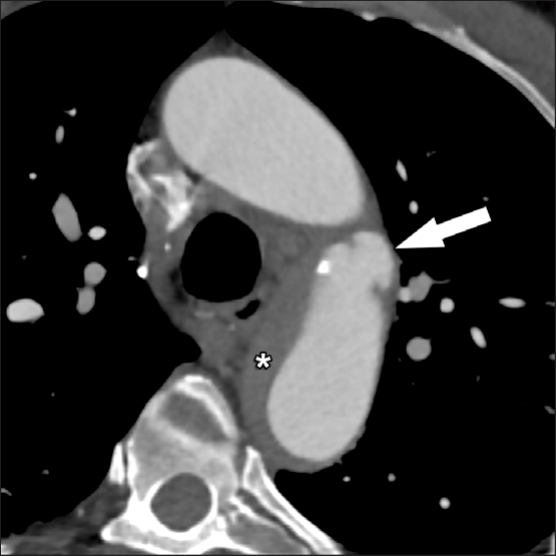
Penetrating atherosclerotic ulcer in a 72-year-old man, with subsequent intramural hematoma (asterisk). This case illustrates again that an intramural hematoma can appear with both a classic dissection and a penetrating ulcer, blurring the distinction between these entities.

Contrary to an acute aortic dissection, a penetrating ulcer has an atherosclerotic origin. In this entity, an atherosclerotic ulcerative focus within a plaque erodes through the intima layer, creating an entry site into the aortic media (Figure 7). Given its origin, this kind of lesion usually is encountered in patients with risk factors like arterial hypertension and diffuse atherosclerosis [7]. The location is also closely linked to arterial segments which are more prone to atherosclerotic degeneration, with the descending aorta far more often involved than other aortic segments. While it can occur in the ascending aorta, it is relatively rare here.

## Cardiac CT in the Emergency Room

The evolving role of CT angiography of the coronary arteries have led to trials and observation studies regarding its possible role in the evaluation of patients with acute chest pain in the emergency department [9,10]. While the application of cardiac CT in an emergency setting does not yet form an integral part of every emergency department, it has been shown to help in the rapid discharge of patients with a low risk for obstructive coronary artery disease [10,11,12].
